# The effect of iron deficiency on the temporal changes in the expression of genes associated with fat metabolism in the pregnant rat

**DOI:** 10.14814/phy2.12908

**Published:** 2016-11-15

**Authors:** Susan M. Hay, Harry J. McArdle, Helen E. Hayes, Valerie J. Stevens, William D. Rees

**Affiliations:** ^1^ The Rowett Institute of Nutrition and Health University of Aberdeen, Foresterhill Aberdeen U.K.

**Keywords:** Development, fetal programming, iron, lipid metabolism, liver, mitochondria

## Abstract

Iron is essential for the oxidative metabolism of lipids. Lipid metabolism changes during gestation to meet the requirements of the growing fetus and to prepare for lactation. The temporal effects of iron deficiency during gestation were studied in female rats fed complete or iron‐deficient diets. Plasma triglycerides were elevated in the iron‐deficient group throughout gestation. There were time‐dependent changes in the triglyceride content of the maternal liver, falling at the midpoint of gestation and then increasing on d21.5. Compared to the control, triglycerides in the maternal liver were not different in the iron‐deficient group prior to pregnancy and on d12.5, but were markedly reduced by d21.5. The abundance of mRNAs in the maternal liver suggests that lipogenesis is unchanged and beta‐oxidation is reduced on d21.5 by iron deficiency. On d21.5 of gestation, the expression of placental lipase was unchanged by iron deficiency, however, the abundance of mRNAs for SREBP‐1c, FABP4 were reduced, suggesting that there were changes in fatty acid handling. In the fetal liver, iron deficiency produced a marked decrease in the abundance of the L‐CPT‐1 mRNA, suggesting that beta‐oxidation is reduced. This study shows that the major effect of iron deficiency on maternal lipid metabolism occurs late in gestation and that perturbed lipid metabolism may be a common feature of models of fetal programming.

## Introduction

Maternal iron deficiency during pregnancy is associated with a range of adverse outcomes in the offspring. Animal studies have shown that the offspring of dams with low iron status have elevated blood pressure (Gambling et al. 2003), altered metabolism (Lewis et al. 2002; Gambling et al. 2003), and poor cognitive and behavioral development (Tran et al. 2012). However, the underlying mechanisms remain elusive. About 0.2–0.5% of the body's iron is found in the active sites of the physiologically essential enzymes of oxidative metabolism, including the cytochromes and iron‐sulfur complexes of the mitochondria (Dallman 1986). It is proposed that during iron deficiency, the electron‐carrying capacity of these enzymes is reduced (Mackler et al. 1984; Evans and Mackler 1985) promoting the use of glucose as the metabolic substrate in place of lipids (Borel et al. 1993; Davis et al. 2012). Changes in lipid metabolism and the expression of associated genes have also been reported in the fetal liver when pregnant rats were fed iron‐deficient diets (Lewis et al. 2001; Zhang et al. 2005). Changes in lipid metabolism or the supply of fatty acids to the developing fetus have also been reported in other models of fetal programming such as high fat (Williams et al. 2014) or folate‐deficient diets (McNeil et al. 2008), suggesting that lipid metabolism may also be involved in the programming mechanism associated with iron deficiency.

The maternal liver has a central role in regulating the lipid supply to the feto‐placental unit. Fatty acids taken up from the gut or released from maternal adipose stores, together with fatty acids produced by de novo synthesis, are processed by the liver and secreted into the peripheral circulation as lipoproteins (Cetin et al. 2009). The pool of triglycerides within the maternal liver reflects the balance between the uptake of fatty acids, de novo lipogenesis, beta‐oxidation, and lipoprotein secretion. Lipids accumulate in the livers of iron‐deficient adults (Ahmed et al. 2012), suggesting that one or more of these processes is sensitive to iron status. In other well‐studied models of nutrition development interactions, such as the methionine‐choline‐deficient diet, intrahepatic lipid accumulation is accompanied by a decrease in the abundance of the mRNA for acyl CoA carboxylase‐1 (Acc1) and an increase in the abundance of the mRNA for liver‐type carnitine palmitoyl transferase‐1 (L‐CPT‐1), as well as corresponding changes in mRNAs for transcriptional regulators such as peroxisome proliferator‐activating receptors (PPAR‐*α* and ‐*γ*) and sterol response element‐binding proteins (SREBP‐1c) (McNeil et al. 2008). The beta‐oxidation of lipids is tightly controlled by the interplay between peroxisomes and mitochondria. The biogenesis of mitochondria and the expression of nuclear encoded mitochondrial genes is tightly regulated by the transcriptional activators PPAR‐gamma‐coactivator‐1 alpha and ‐beta (PGC‐1*α* and PGC‐1*β*) and the mitochondrial transcription factor A (TFAM) (Archer 2013). One of the aims of this study was to investigate the effects of iron deficiency on the intrahepatic triglyceride pool of the maternal liver and the expression of genes coding for enzymes associated with lipid metabolism during gestation.

The fetus derives its fatty acids from the peripheral circulation. Free fatty acids from the maternal plasma, together with those released from lipoproteins by placental lipase action, are taken up by fatty acid transporters in the placenta (Duttaroy 2009). The availability of fatty acids in the maternal circulation may therefore regulate placental transport and metabolism via transcription factors including PPAR‐*α*, PPAR‐*γ,* and SREBP‐1c within the placenta (Duttaroy 2006). As prepregnancy iron deficiency modifies lipid concentrations, gestational iron deficiency may produce changes in the abundance of key mRNAs associated with placental lipid transporters and function. Finally, we have also studied the abundance of mRNAs associated with lipid metabolism in the fetal liver.

## Methods

### Animals

All experimental procedures were approved by the ethical review committee of the Rowett Research Institute and conducted in accordance with the UK Animals (Scientific Procedures) Act, 1986. Animals used in this study have been described previously (Gambling et al. 2009). Briefly, weanling rats were fed control diets for 2 weeks prior to being randomly allocated into two groups. The animals, with a mean body weight of 91.6 ± 8.5 g, were fed semisynthetic diets containing 20% w/w dried egg albumin, 7.5% w/w groundnut oil, with FeSO_4_ providing either 50 mg Fe/kg diet (control) or 7.5 mg Fe/kg diet (FeDef) as described previously (Gambling et al. 2003). After feeding the experimental diets for 4 weeks, the animals (weighing 212.6 ± 10.8 g [control] and 204.3 ± 14.8 g [FeDef]) were mated with normal males and then continued to be fed the same experimental diets until d0.5, d12.5, or d21.5 of gestation, when dams were fasted for 8 h, anesthetized with isoflurane and blood samples were taken from the heart before the animals were killed by cervical dislocation. Tissues were rapidly removed, frozen in liquid nitrogen, and stored at −80°C until required.

### qRT‐PCR

Frozen tissue samples (30 mg) were crushed before DNase treated total RNA was isolated using the Qiagen RNeasy mini kit (Qiagen, Manchester UK) following the manufacturer's instructions. The RNA was checked for integrity and quantified using an Agilent 2100Bioanalyser (Agilent Technologies, Stockport, Cheshire, UK). Samples of 200 ng total RNA with an RNA integrity number >8 were reverse transcribed with the TaqMan Reverse Transcription Reagents Kit (Applied Biosystems, Warrington, Cheshire, UK) primed with random hexamers following the manufacturer's instructions. The products were diluted to give a final concentration equivalent to 1 ng RNA per μL and 5 μL aliquots were used for the PCR reactions which were carried out with either the SYBR Green real time PCR kit with primer sequences described previously (Lavrentyev et al. 2004; Maloney et al. 2007; McNeil et al. 2008) or TaqMan^®^ Gene Expression Assays (Applied Biosystems) for transcription factor A, mitochondrial (TFAM: Rn00580051_m1), peroxisome proliferator‐activated receptor gamma coactivator 1‐beta (Ppargc 1b: Rn00598552_m1), fatty acid transporter (Slc27a1: Rn00585821_m1), endothelial lipase (Lipg:Rn01523950_m1), and fatty acid‐binding protein 4 (FABP4: Rn00670361_m1). The qPCR reactions used the standard protocol provided by the manufacturer. The abundance of cDNAs were measured relative to the 18S ribosomal RNA (Cat no 4319413E, Eukaryotic 18S rRNA Endogenous Control [VIC^®^/MGB probe, primer limited]; Applied Biosystems) and relative target quantity was calculated from a standard curve in order to facilitate comparisons between the SYBR Green and TaqMan chemistries.

### Triglyceride analysis

Serum triglyceride concentrations were measured using TAG reagent (ThemoElectron Triglyceride Kit; Lab Medics Ltd) with a Konelab selective chemistry analyzer (Labmedics Ltd, Salford, Manchester, UK).

Hepatic triglyceride concentrations were measured in chloroform extracts as detailed previously (McNeil et al. 2008). Briefly, approximately 0.2 g tissue was homogenized in 1 mL ice‐cold 0.145 mol/L NaCl and extracted with 10 mL chloroform:methanol (2:1) and the chloroform phase transferred to a clean tube. The aqueous phase was washed with chloroform and, the extracts were combined, dried and the lipids dissolved in 5 mL absolute ethanol. Replicate 0.01 mL samples of the ethanol suspension were mixed with 0.25 mL TAG reagent, and incubated for 20 min at room temperature before measuring the absorbance at 510 nm. TAG concentrations in the samples were determined from a standard curve.

### Statistical analysis

Data are presented as mean ± SEM and analyzed using Genstat (12th/13th edition, VSN International Ltd, Hemel Hempstead, UK). Differences between groups were assessed by Students t‐test or by two‐way ANOVA with gestational age and diet as factors. When the effects were significant, means were compared post hoc by Fisher's unprotected least significant difference test. Correlation between parameters was assessed using the linear regression function of Genstat. Statistical significance was set at *P* < 0.05 unless indicated otherwise.

## Results

### Maternal liver

The body weight of the animals was similar until d19.5 of gestation (Control 332.0 ± 5.3 g vs. FeDef 323.4 ± 7.9 g; *P* = 0.37), however, by d21.5 of gestation, animals in the FeDef group were approximately 12% lighter (Control 359.3 ± 8.2 g vs. FeDef 315.5 ± 18.9 g; *P* = 0.032). The pups at d21 of gestation were smaller in the FeDef group (4.1 ± 0.1 g) when compared to the control (4.8 ± 0.1 g, *P* < 0.001) (Gambling et al. 2009). The dams hematocrit, which was not different at mating (Control 40.8 ± 1.4% vs. FeDef 40.2 ± 2%), tended to be different at 12.5 days (Control 37.0 ± 0.4% vs. FeDef 36.0 ± 1.0%; *P* = 0.068) and was different by d21.5 of gestation (Control 35.0 ± 0.8% vs. FeDef 27.2 ± 0.8%; *P* < 0.001). The d21.5 fetal hematocrit was also lower (Control 29.0 ± 1% vs. FeDef 22.0 ± 1.0%; *P* < 0.001). The iron content of the maternal liver on d21.5 of gestation was also reduced from 488.5 ± 32.7 μg/g dry weight in the control to 74.3 ± 5.4 μg/g dry weight in the FeDef group (*P* < 0.001).

**Figure 1:**
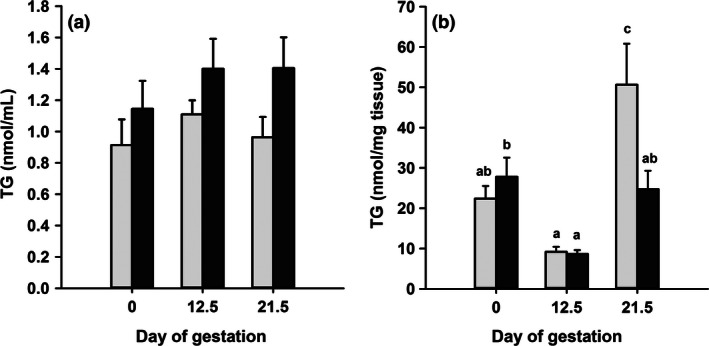
Maternal plasma and liver triglyceride. (A) Plasma triglyceride (TG) concentration in female rats fed control (light bars) or iron‐deficient diets (dark bars) on different days of gestation. Data are presented as mean + SEM (*n* = 6). Data analyzed by two‐way ANOVA gestational age n.s., diet *P* = 0.014 and gestational age.diet n.s. (B) Triglyceride (TG) concentrations in maternal liver during gestation. Data are presented as mean + SEM (*n* = 6). Data analyzed by two‐way ANOVA gestational age *P* < 0.001, diet n.s. and gestational age.diet *P* = 0.031. ^a,b^Bars with different letter superscripts are significantly different Fisher's LSD test.

Fasting plasma triglyceride concentrations (Fig. 1A) remained constant throughout gestation, but were increased by 20–40% compared to the control at all stages of gestation in the iron‐deficient animals (diet *P* = 0.014). Triglyceride concentrations in the intrahepatic pool of the maternal liver (Fig. 1B) changed substantially during the course of pregnancy (gestational age *P* < 0.001). In the animals fed the complete diet, intrahepatic triglyceride concentrations at the midpoint of gestation (d12.5) were about half of those of animals on d0.5 of gestation but then increased, such that by the end of gestation (d21.5), concentrations were approximately twofold higher than at the start. Intrahepatic triglyceride concentrations in the iron‐deficient group were not different from the controls on either d0.5 or d12.5 of gestation. The major effect of iron deficiency was to prevent the increase in intrahepatic triglyceride which occurred late in gestation in the control group. On d21.5 of gestation, concentrations of triglyceride in the livers of animals in the iron‐deficient group were similar to those on d0.5 of gestation and approximately twofold less than in the control.

**Figure 2:**
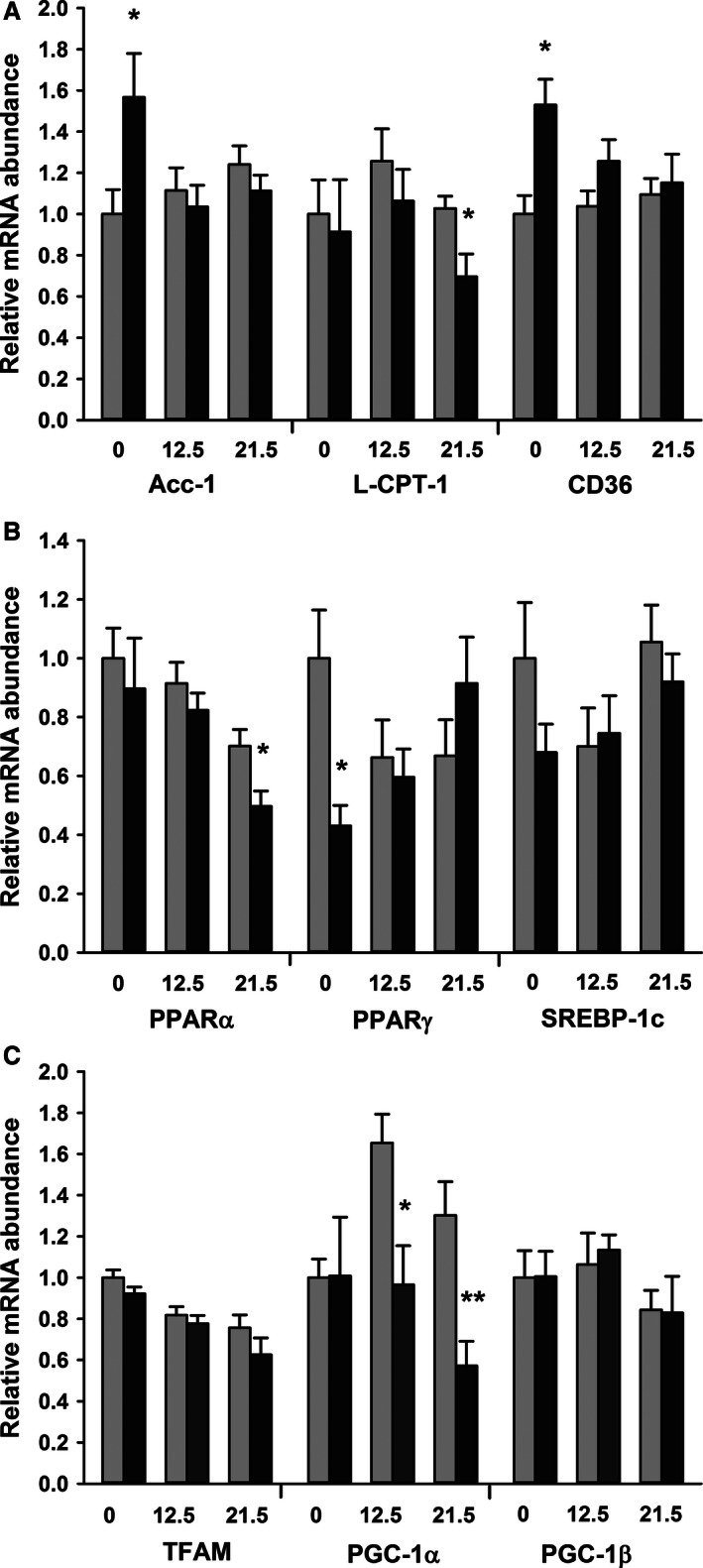
Temporal changes in relative mRNA abundance of genes associated with fat metabolism in maternal liver. Temporal changes during gestation in the abundance of mRNAs in the liver of female rats fed diets containing sufficient iron (light bars) or iron deficient (dark bars). Data are presented as mean + SEM (*n* = 6) and expressed in arbitrary units relative to the 18S ribosomal RNA and normalized to the levels of the prepregnant animals fed the control diet. *P < 0.05 and **P < 0.01 compared to control by Student's *t*‐test. (A) mRNAs coding for enzymes of lipid metabolism. Data analyzed by two‐way ANOVA; Acc1 gestational age n.s., diet n.s. and gestational age.diet *P* = 0.031; L‐CPT1 gestational age n.s., diet n.s. and gestational age.diet n.s.; CD36 gestational age n.s., diet 0.009 and gestational age.diet n.s. (B) mRNAs coding for transcriptional activators. Data analyzed by two‐way ANOVA; PPAR‐*α* gestational age 0.002, diet n.s. and gestational age.diet n.s.; PPAR‐*γ* gestational age n.s., diet n.s. and gestational age.diet *P* = 0.032 and SREBP‐1c gestational age n.s., diet n.s. and gestational age.diet n.s. (n.s. = *P* > 0.1.). (C). mRNAs coding for mitochondrial transcription. Data analyzed by two‐way ANOVA; TFAM gestational age *P* < 0.001, diet n.s. and gestational age.diet n.s. PGC‐1*α* gestational age n.s., diet *P* = 0.002 and gestational age.diet n.s. PGC‐1*β* gestational age n.s., diet n.s. and gestational age.diet n.s.

The relative abundances of the mRNAs associated with fatty acid synthesis, transport, and oxidation in the maternal liver were influenced by both iron status and stage of gestation (Fig. 2A). The mRNA coding for Acyl‐CoA carboxylase (Acc‐1) which is the rate‐limiting step in de novo lipogenesis remained constant throughout gestation in the control animals. The Acc‐1 mRNA on d0.5 of gestation was 1.6 times as abundant in the livers of animals fed the Fe‐deficient diet compared to the control, however, these differences disappeared as gestation progressed (gestational age.diet interaction *P* = 0.030). In contrast, on d0.5 of gestation, iron deficiency did not change the relative quantities of the mRNA coding for L‐CPT‐1, a regulator of fatty acid oxidation, whereas by d21.5 of gestation, it was reduced by approximately 30% in the livers of dams fed the Fe‐deficient diet compared to the control (*P* = 0.023 by one‐way ANOVA). The abundance of the mRNA for CD36, remained constant throughout gestation, but was increased in the animals fed the Fe‐deficient diet compared to the controls (diet *P* = 0.009).

The abundance of the mRNAs associated with genes which regulate fatty acid synthesis and beta‐oxidation were also influenced by iron status and stage of gestation (Fig. 2B). The abundance of the mRNA coding for PPAR‐*α* declined by 30–40% as gestation progressed (gestational age *P* = 0.002), however, there was no effect of diet and no diet.gestational age interaction. The mRNA coding for PPAR‐*γ* was unaltered as gestation progressed but there was an interaction between stage of gestation and diet (diet.gestational age *P* = 0.034). On d0.5 of gestation, the relative abundance of the PPAR‐*γ* mRNA in the iron‐deficient group was approximately half that of the controls, but by d21.5 of gestation, expression had increased such that it was approximately 30% higher in the iron‐deficient group compared to the controls. The abundance of the mRNA for SREBP‐1c was unaffected by either the stage of gestation or the composition of the diet.

The temporal changes in the abundance of mRNAs associated with the regulation of mitochondrial activity in the maternal liver of pregnant animals are shown in Figure 2C. The expression of TFAM, the main regulator of nuclear mitochondrial transcripts was unaffected by either the stage of gestation or the composition of the diet. In the control group, the abundance of the mRNA for PPAR‐gamma coactivator 1*α* (PGC1‐*α*) tended to increase as gestation progressed (time *P* = 0.09 by 2‐way ANOVA), whereas the abundance declined in the Fe‐deficient group, such that by d21 of gestation, there was a marked reduction with the abundance only approximately 40% of the control group (diet *P* = 0.002 by two‐way ANOVA). There was no change in PPAR‐gamma coactivator‐1*β* (PGC‐1*β*), with either time or diet.

### Placenta

**Figure 3:**
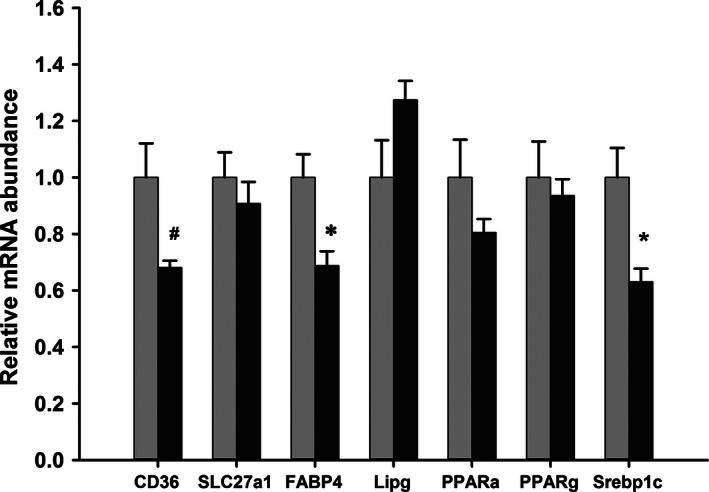
Relative mRNA abundance in the placenta on d21.5 of gestation. The abundance of mRNAs in the d21.5 placentae in pregnant rats fed control (light bars) or iron‐deficient (dark bars) diets. Data are presented as mean + SEM (*n* = 6) and expressed in arbitrary units relative to the 18S ribosomal RNA and normalized to the levels of the animals fed the control diet. Data analyzed by Students *t*‐test, *P < 0.05. ^#^ *P* < 0.1

Iron status altered the abundance of mRNAs associated with lipid transport in the placenta on d21.5 of gestation (Fig. 3). Compared to the controls, the mRNA coding for FABP4 was reduced (*P* = 0.017) and the fatty acid transporter CD36 tended (*P* = 0.057) to be lower in the Fe‐deficient animals. In contrast, the mRNAs for endothelial lipase (Lipg) and the fatty acid transporter SLC27a1 (FATP1) were not different. The abundance of the mRNAs for PPAR‐*α* and PPAR‐*γ* were unchanged, but there was a decrease in the mRNA coding for SREBP‐1c in the iron‐deficient group (*P* = 0.020) and this showed a linear positive correlation with the abundance of the mRNA for CD36 (*R* ^2^ = 0.81). The abundance of the SREBP mRNA also showed a weak negative correlation with plasma triglyceride concentrations (*R* ^2^ = 0.27).

### Fetal liver

**Figure 4:**
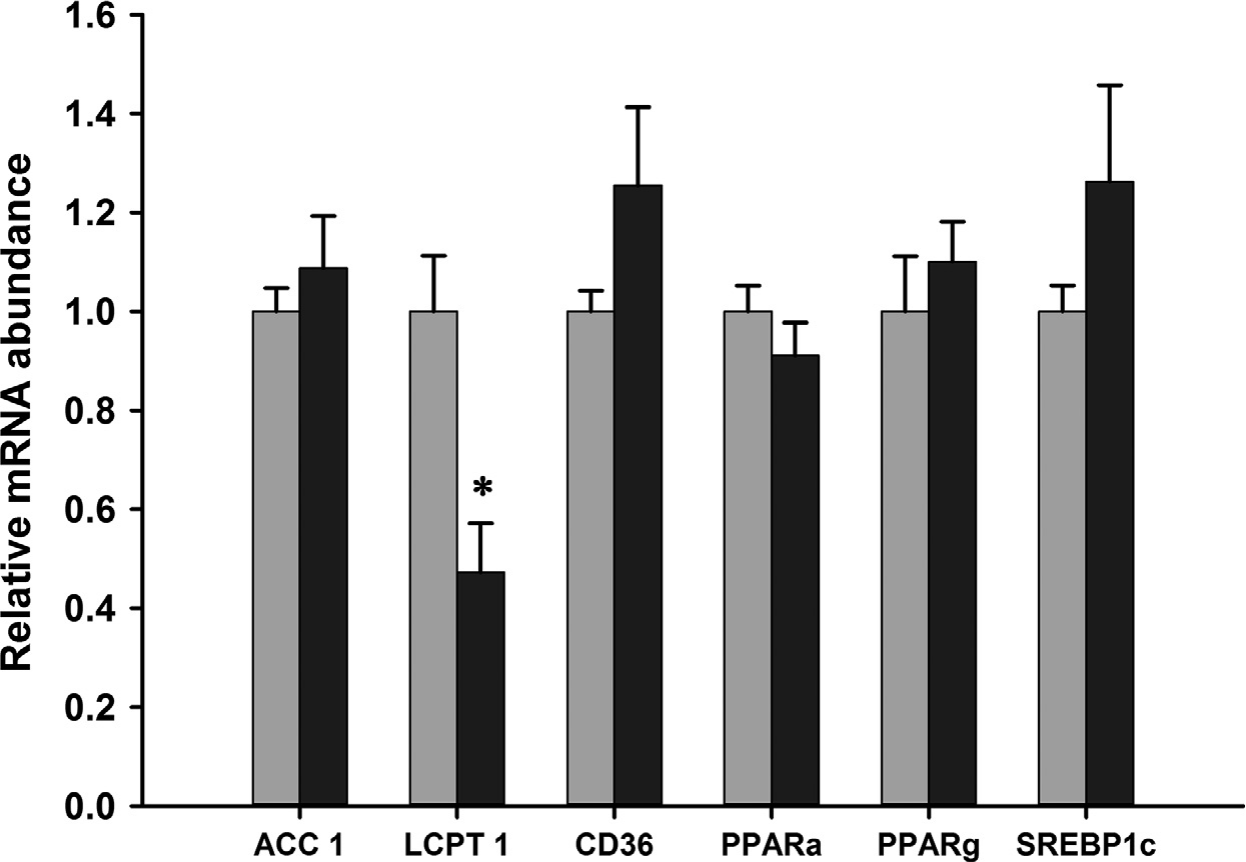
Relative mRNA abundance in the fetal liver on d21.5 of gestation. The relative abundance of mRNAs in the fetal liver on d21.5 of gestation in rats fed control (light bars) or iron‐deficient (dark bars) diets. Data are presented as mean + SEM (*n* = 6) and expressed in arbitrary units relative to the 18S ribosomal RNA and normalized to the levels of the animals fed the control diet. Data analyzed by Students *t*‐test, *P < 0.05.

In the fetal liver on d21.5 of gestation (Fig. 4), the abundance of the mRNAs coding for Acc1 and CD36 were similar in both diet groups, but there was a marked reduction of approximately 50% in the mRNA coding for L‐CPT‐1 in the iron‐deficient group. The abundance of the mRNAs associated with regulatory genes PPAR‐*α*, PPAR‐*γ,* and SREBP‐1c in fetal livers were unaffected by the iron content of the dams diet. The abundance of TFAM, PGC1‐*α,* and PGC1‐*β* mRNAs in the fetal livers on d21.5 of gestation was unaffected by iron deficiency (data not shown).

## Discussion

Lipids are of fundamental importance for the developing fetus, both as substrates for energy metabolism and as structural components of cellular membranes. During gestation, there are substantial changes in lipid turnover to meet the additional and changing metabolic demands of the fetus. During the first part of pregnancy, the rat is hyperphagic, storing fat to provide a reserve which when mobilized at the end of gestation provides fatty acids for fetal and mammary development (Herrera 2002). The triglyceride pool within the dams liver reflects these changes, first declining around d12.5 as body fat accumulates in the adipocytes and then increasing again on d21.5 just before birth as a consequence of a reduction in beta‐oxidation of fatty acids and an increase in fatty acid esterification (Wasfi et al. 1980). This study shows that the handling of triglyceride within the maternal liver and also the abundance of the mRNAs regulating its metabolism are changed by iron deficiency with the most pronounced effects occurring in the later stages of gestation.

Around the time of conception, iron deficiency tends to increase plasma and intrahepatic triglyceride concentrations and is accompanied by an increase in the abundance of the Acc‐1 mRNA which is associated with lipogenesis. These findings are consistent with an increase in the incorporation of glucose into polar lipids and suggest a shift from fat to glucose as the preferred substrate for oxidative metabolism, results which are similar to those previously reported in the male rat (Davis et al. 2012). As gestation progresses, this modest effect of iron deficiency continues up to the midpoint, where the data suggest that there is no difference in either the intrahepatic fat pool or in the mRNAs associated with fat metabolism. At this stage of gestation, the fetal tissues represent a relatively small portion of the total body mass and when coupled with an increase in the limited intake of iron due to hyperphagia, it is probable that the impact on the dam's iron status is very limited – a suggestion supported by the modest tendency for the hematocrit to be lower at this time point.

The effects of iron deficiency on fat metabolism in the maternal liver are most pronounced in the latter stages of gestation when the degree of iron deficiency, judged by hematocrit, is also at its greatest. This is the period when the increase in intrahepatic triglyceride concentrations which occurs in the control animals is markedly suppressed in the iron‐deficient group. This reduction to about 40% of the control values cannot be explained by a decrease in lipogenesis, since by d21.5, Acc‐1 mRNA is present at similar levels in both control and iron‐deficient animals. Furthermore, the abundance of the L‐CPT‐1 mRNA is decreased (*P* = 0.023 by one‐way ANOVA), suggesting reduced transfer of fatty acids to the mitochondria. As in the mouse (Sweeney et al. 2006), there is a decrease in the abundance of the PPAR‐*α* mRNA as gestation progresses, suggesting that it has a role in reducing beta‐oxidation. However, the change in the abundance of the PPAR‐*α* mRNA in the iron‐deficient group is small relative to the iron‐sufficient control group, suggesting that it does not mediate the effects of iron deficiency. The mRNA for L‐CPT‐1, the transcription of which is partly dependent on PPAR‐*α* is reduced in the liver on d21.5, suggesting a role for PPAR‐*α*‐independent mechanisms, for example, those involving PGC‐1*α* (Song et al. 2010). The absence of an effect of iron deficiency on the abundance of the SREBP‐1c mRNA suggests that it is not a part of the response to iron deficiency. When lipids accumulate in the maternal liver of pregnant rats fed methyl‐deficient diets (i.e., low in folic acid, choline, and methionine), there is an increase in the abundance of the PPAR‐*γ* mRNA (McNeil et al. 2008). This is in keeping with the general observation that PPAR‐*γ* is overexpressed in the livers of steatotic animals and that hepatocyte‐specific PPAR‐*γ* knockouts have decreased hepatic lipid accumulation (Morán‐Salvador et al. 2011). However, in the iron‐deficient pregnant animals, PPAR‐*γ* expression changes in the absence of changes in intrahepatic lipid. Before pregnancy is established, iron deficiency decreases the abundance of the PPAR‐*γ* mRNA in the absence of a change in intrahepatic triglyceride and on d21.5 of gestation, the mRNA increases in abundance despite the intrahepatic triglyceride concentration being similar to the prepregnancy levels. This suggests that the changes in PPAR‐*γ* expression are not a response to the accumulation of lipid and that an alternative mechanism is involved in iron deficiency.

Late in gestation, there is also a marked reduction in the abundance of the mRNA coding for PGC‐1*α*, a transcriptional coactivator that along with PPAR‐*γ* regulates pathways linked to energy homeostasis. In the liver, PGC‐1*α* is induced during fasting, when the use of glucose is reduced and the *β*‐oxidation of fatty acids is increased (Herzig et al. 2001; Yoon et al. 2001). The fall in the abundance of PGC‐1*α* may be linked to the change in L‐CPT‐1 abundance and reflect a reduction in beta‐oxidation. However, an alternative explanation may reflect the role of PGC‐1*α* in the regulation of heme metabolism. PGC‐1*α* is involved in regulating the transcription of 5‐aminolevulinate synthase, the rate‐limiting enzyme in hepatic heme biosynthesis (Handschin et al. 2005; Wu et al. 2009). There is a striking parallel between the iron status of animals in the deficient group, which falls only in the d21 maternal liver (Gambling et al. 2009), and the expression of PGC‐1*α*, suggesting that this may be an important link between nutritional status and heme biosynthesis, as suggested by the correlation on d21.5 of gestation between PGC‐1*α* mRNA abundance and both hematocrit (*R* ^2^ = 0.38, P = 0.021) and the iron content of the maternal liver (*R* ^2^ = 0.42, *P* = 0.012).

The intrahepatic lipid pool is the product of fatty acid uptake, synthesis, oxidation, and export. Of these factors, a reduced flow of lipids into the maternal liver, a consequence of either reduced lipolysis by adipocytes or a decrease in the uptake from the gut is a possible explanation for the reduction in intrahepatic triglyceride in the latter stages of gestation. There is evidence that iron or transferrin stimulates lipolysis in isolated adipocytes (Rumberger et al. 2004), although direct evidence for an impact of deficiency is lacking. The gut may also be a possible target as gene array studies have shown that lipid metabolism is altered in the duodenum of iron‐deficient rats (Collins 2008) and studies in vitro have shown that the assembly and secretion of lipoproteins by enterocytes is dependent on the iron status (Courtois et al. 2000). The possibility that iron deficiency may limit fatty acid uptake, particularly the long‐chain polyunsaturated fatty acids, is worth further investigation as one of the major adverse effects of maternal iron deficiency is cognitive impairment of the offspring (Georgieff 2011).

The placenta which is the interface between the maternal and fetal circulation, has the potential to respond to changes in the lipid supply. For example, in the growth restricted human fetus, there are decreases in the abundance of endothelial lipase (Lipg) mRNA and increases in lipoprotein lipase mRNA (Lpl) (Gauster et al. 2007). Although triglyceride concentrations in the maternal plasma are about 40% higher in the iron‐deficient animals throughout gestation, the absence of any change in the abundance of the mRNAs coding for lipase, suggests that this route of uptake is unchanged and that lipase activity is responsive to fetal growth as opposed to nutrient limitation. As lipases may be resent in inactive forms, it is possible that changes in mRNA levels do not reflect the enzyme activity (Lindegaard et al. 2005). However, the reduced abundance of the FABP4 fatty acid‐binding protein mRNA and a tendency for a similar reduction in the CD36 mRNA, both of which code for proteins which transport free fatty acids within the placenta, does suggest that the supply of fatty acids may be compromised in some way. The decrease in the abundance of SREBP‐1 mRNA, with no change in PPAR‐*α* and PPAR‐*γ*, in the placenta of the iron‐deficient animals also suggests a change in free fatty acid turnover as it has been suggested that placental SREBP‐1 controls the expression of enzymes responsible for the endogenous synthesis of saturated and monounsaturated fatty acids (Weedon‐Fekjaer et al. 2005; Duttaroy 2009). The long‐chain fatty acids produced by these enzymes are key components of membrane phospholipids, suggesting that while the bulk transfer of fatty acids is unaffected, iron deficiency may have specific effects on the availability of particular fatty acids.

With the exception of a decrease in the abundance of L‐CPT‐1 mRNA, suggesting that the oxidation of fatty acids is reduced in iron deficiency, there was no evidence for other changes in lipid metabolism in the fetal liver. Previous studies have shown that the expression of SREBP‐1c and its associated target genes was reduced in the fetal liver when dams were fed an iron‐deficient diet (Zhang et al. 2005). The discrepancy between the study of Zhang et al. and the present results may be the consequence of the substantial differences in the experimental diets; 3 mg Fe/kg diet compared to 7.5 mg Fe/kg in the present experiment and 5.2% w/w fat compared to 10%w/w fat in the present experiment. The impact of this apparent interaction between iron and fat in the diet of the pregnant animal is worthy of further investigation. Overall, the data from the present experiment suggest that fetal lipid metabolism is largely protected, however, the decrease in L‐CPT‐1 abundance does suggest that fat oxidation is reduced in the fetal liver. Carnitine, used by L‐CPT‐1 for the production of acylcarnitines, is synthesized by a pathway containing two iron‐dependent enzymes (Vaz and Wanders 2002). While iron deficiency does not affect the concentration of carnitine in the liver, there is evidence for the impaired transport of carnitine to heart and skeletal muscle in female rats (Bartholmey and Sherman 1986). The reduced availability of carnitine late in gestation may be related to the reduced expression of L‐CPT‐1 in the livers of both dam and fetus on d21.5 of gestation.

### Perspectives and significance

This study shows that iron deficiency has an impact on the temporal changes in lipid metabolism in the maternal liver during gestation. The effect is most pronounced late in gestation – a period when maternal lipid metabolism is changing to support mammary development and prepare for lactation. This raises the possibility that iron deficiency may go on to affect the offspring via changes in milk production. Changes in lipid metabolism during gestation are not unique to iron deficiency; a number of other well‐characterized models of fetal programming are also associated with abnormal lipid metabolism. For example, maternal diets with imbalances in each of the macronutrients, fat (Zhang et al. 2009), carbohydrate (D'Alessandro et al. 2012), and protein (McNeil et al. 2007), all increase triglyceride concentrations in the maternal circulation and affect intrahepatic triglycerides. This is also the case for micronutrient deficiencies such as folic acid (McNeil et al. 2008; Maloney et al. 2009). These observations suggest that perturbed lipid metabolism during gestation may be a common mechanism through which the maternal diet programs the offspring's physiology during fetal development.

## Conflict of Interest

None declared.
